# Parent-led Communication Therapy for Young Bilingual Autistic Children: A Scoping Review

**DOI:** 10.1007/s10803-024-06478-0

**Published:** 2024-08-11

**Authors:** Sarah Oudet, Katie Howard, Christina Gilhuber, Marie Robert, Joanna Zimmerli, Napoleon Katsos, Stephanie Durrleman

**Affiliations:** 1 University of Fribourg (Science and Medicine), Fribourg, CH Switzerland; 2 University of Exeter (Education), Exeter, UK; 3 Illinois State University (Special Education), Normal, USA; 4 Agence Régionale de Santé (Hauts-de-Seine), Nanterre, France; 5 RCSLT-HCPC (SLT), London, UK; 6 Paris, France; 7 University of Cambridge (Experimental Pragmatics), Cambridge, UK

**Keywords:** Autism, Bilingualism, Parent-led therapy, Early years, Scoping review

## Abstract

A scoping review of the literature was undertaken using JBI guidelines to map the evidence of parent-led therapy (PLT) for young autistic children (≤ 6 years) raised in bilingual environments. Reviewers used Covidence to screen located sources. Sixteen papers met inclusion criteria. A strong acceleration of reports of PLT for young autistic children measured in bilingual environments was observed, with 93.8% of papers (n = 15) published since 2015. Reporting of participants’ language environments (home language(s)/L1s and societal language(s)/L2s) was inconsistent. A large majority of these studies, 87.5% (n = 14) were conducted in North America or in collaboration with a North American institution. Diverse PLT programs and methodologies were identified. There is variation in demographic information collected and outcomes reported. Evidence gaps in the literature are identified and the value of undertaking systematic review on this topic is considered. This scoping review points to the necessity of further empirical research and practice that centres parents in early and specific support for autistic children raised in bilingual environments. Suggestions for improving reporting standards of language profiles are provided.

In an increasingly globalised world, where many countries have established more than one national language, bilingualism is the norm for more than half the world’s population (Jaumont, 2017). A growing body of evidence about bilingually exposed autistic^1^ children is emerging with consensus that bilingualism^2^ does not delay or cause detriment to the child’s language development (Hambly & Fombonne, 2012; Lund et al., 2017; Ohashi et al., 2012; Petersen et al., 2012; Uljarević et al., 2016). Bilingual autistic children have been shown to perform similarly to, if not better than, their monolingual peers in measures of expressive and receptive vocabulary (Dai et al., 2018; Hastedt et al., 2023), structural language and pragmatic ability (Reetzke et al., 2015), gesture use (Zhou et al., 2019), and social communication skills (Hastedt et al., 2023). Specifically, autistic children who benefited from simultaneous bilingual exposure (from infancy) have been shown to have stronger social interaction skills than their sequentially exposed (post-infancy) peers (Hambly & Fombonne, 2012). Moreover, studies have found positive influence of bilingual exposure for cognitive and communication skills of autistic children, such as executive functions, including attention (Gonzalez-Barrero & Nadig, 2019; Sharaan et al., 2021), inhibitory control (Montgomery et al., 2022), theory of mind (Cummings et al., 2024; Peristeri et al., 2021), and numerical operations (Vanegas, 2019). Social-emotional benefits have also been identified for bilingual autistic children, including the development of multicultural identities (Jegatheesan, 2011), preservation of heritage (Yu, 2013), and enriched family relationships (Hampton et al., 2017; Howard et al., 2020; Kay-Raining Bird et al., 2016a).

In parallel to the documentation of a so-called “bilingual advantage”, research has shown negative effects for both the autistic child and their family when they, from bilingual families, are raised monolingually (Beauchamp & MacLeod, 2017). Restricting children’s language exposure to the dominant language, often English, may impede the enhancement or even formation of familial relationships (Ijalba, 2016). This may reduce communication opportunities for the child, which, while disadvantageous for all children, may be particularly damaging to autistic children, for whom social-emotional relationships may be especially challenging (APA, 2013). The number of places and interlocuters with whom children use a language is a significant predictor of their skills in that language (Sun et al., 2022). Access to a heritage language has essential implications for the formation of cultural identity in children (Imbens-Bailey, 1996; Oh & Fuligni, 2010) and well-being, including bimodal bilingual environments (signed languages) (Reynolds, 2018).

The value of early intervention or specific support during the early years^3^ focused on developing communication skills for autistic children is widely accepted. However, therapy is often not received until after a diagnosis is delivered. Whitehouse et al (2021) identified that pre-emptive support for young children during infancy and the early years can improve outcomes and reduce the likelihood of an autism diagnosis at age three (Whitehouse et al., 2021). Vivanti et al (2022) identified that children who spend more time directly interacting with and paying attention to people prior to receiving structured and specific support experience more social-communicative gains in inclusive preschool settings (Vivanti et al., 2022). One way to maximise chances of specific support being implemented as early as possible is to empower parents to play an active role in therapy. Parent–child interactions are critically important for autistic children, or those at high likelihood of receiving a diagnosis, for whom social communication is more difficult and limited (Zlomke & Jeter, 2019). This is particularly true during the early years, when children are likely to spend more time in their homes and neighbourhoods than other settings (Hyman et al., 2020; Zwaigenbaum et al., 2019). A growing body of evidence is emerging in support of parent-led therapy (PLT) for children with developmental difficulties, including autism (Dawson et al., 2010; Green et al., 2010; Heidlage et al., 2020; Rakap & Rakap, 2014; Roberts & Kaiser, 2011; Wetherby et al., 2014; Wong et al., 2015). However, the inclusion of bilingually exposed children in communication-focused PLT studies is relatively novel. Even less is known about the impact of monolingually validated parent-led communication programs on autistic children in bilingual environments.

PLT is even more critical for autistic children from culturally and linguistically diverse (CaLD) families, for whom access to dual/multi-language and communication support is more difficult (Oudet et al., 2022). Recent studies suggest benefits associated with specific support delivered in the heritage language rather than solely in the majority language (Lim et al., 2019), and have called for greater provision of resources for bilingual parents of autistic children (Davis et al., 2021; Gréaux et al., 2020; Howard et al., 2020). Despite a plethora of evidence demonstrating the importance of maintaining bilingualism for children on the autism spectrum, there remain many barriers to accessing learning and communication support for CaLD families (Davis et al., 2021; de Valenzuela et al., 2016; Marinova-Todd et al., 2016; Pesco et al., 2016). While studies have called for full inclusion policies that include increased bilingual support for autistic children and their families (Beauchamp & MacLeod, 2017; de Valenzuela et al., 2016; Kay-Raining Bird et al., 2016b; Lim et al., 2018), wide implementation of such support has yet to manifest (Oudet et al., 2022).

## The Review

United Nations policy papers call for the recognition and protection of communication rights of autistic children, particularly those from CaLD backgrounds (Gréaux et al., 2020). Clinical guideline papers have highlighted the need for active parent involvement in therapeutic support for their autistic children (Zwaigenbaum et al., 2015). More recently, PLT has been specifically emphasised as an important part of early care and support targeted at young autistic children and their families (Hyman et al., 2020; Lim et al., 2018). However, no review has yet synthesised existing research on PLT to support the communication needs of bilingual autistic children.

Bilingual parents report that raising an autistic child is challenging and stressful (Giovagnoli et al., 2015; Ilias et al., 2018; Lai et al., 2015), and express confusion and anxiety when making language decisions for their children (Drysdale et al., 2015; Howard et al., 2020; Kay-Raining Bird et al., 2016a, 2016b, 2016c). PLT is particularly interesting for children raised in bilingual environments, as empowering parents to implement strategies immediately to their child in their L1 may address difficulties including the reported lack of cultural and linguistic diversity in the speech-language therapy profession, and non-inclusive clinical tools (Davis et al., 2023). Alarmingly little is known about the feasibility, accessibility, and effectiveness of communication-focused PLT for autistic children in bilingual environments. Given the apparent disconnect between public policy and clinical guidelines, research evidence, and reported clinical practice, as well as the known and complex barriers to accessing appropriate therapy for CaLD families (Wallace-Watkin et al., 2023), this review serves to formally acknowledge and report existing research gaps.

## Aim

In light of the relatively recent emergence of this field of study, this scoping review aims to provide an outline of the volume and nature of current evidence on PLTs for autistic children and the communication outcomes of such programs that have included children in bilingual environments. Scoping reviews provide broad and exploratory reviews of available data, and allow investigators to map the data across disciplines, including grey literature, and identify gaps in the evidence base (Arksey & O’Malley, 2005). This scoping review does not attempt to critically appraise the included studies, nor provide a quality assessment of the PLT programs studied, which is the purview of a systematic review. In kind, this scoping review does not intend to evaluate nor interpret the evidence collected, for which a literature review would be appropriate.

This review will allow for the origins of published literature on PLT targeting communication skills for young autistic children in the bilingual context to be identified. This includes the countries in which the research took place, specific language environments studied, and socio-economic demographics included. For the purposes of this study, PLT programs will include both parent-mediated (active parent-training or “coaching” provided by a clinician) and self-directed services (published manuals/guidebooks used by parents independently). The use of the term “parent” in this study includes legal guardians and key caregivers of the child in question. The use of the term “bilingual environment” in this study includes exposure to two or more languages across daily settings. No ethical review was required for this study.

## Developing the Research Question

The initial research question posed was: What parent-led communication therapy programs are available for families of autistic children? This question was used to conduct a pilot literature search to gain up-to-date information about specific parent-led communication therapies that are available to support the early communication development of autistic children. Search terms were identified at five levels (Table 1). A pilot search was conducted using the University of Fribourg library engine with search levels 1–4 produced 18,684 results. A similar pilot search with all 5 search levels, that is, including the bilingual search level, produced 1,652 results. This highlighted that, while broadly recommended, particularly for CaLD families, PLT in the bilingual environment has had comparatively little examination. It is therefore not well established whether PLT programs are effective in the bilingual context.

**Table 1:** Levels of search terms

Level 1	Developmental disorder	“development* disorder” OR autis* OR “autism spectrum” OR “asd”
Level 2	Early intervention	“early intervention” OR p?ediatric OR child* OR infant OR toddler OR pre?school OR “young child*” OR “early years”
Level 3	Communication therapy	“language acquisition” OR “language development*” OR “social?comm*” OR “pragmatic*” OR “communication” OR “interaction*”
Level 4	Parent-led	“parent-led” OR “self-directed” OR “parent-mediated” OR “parent-implemented” OR “parent-delivered” OR “parent training” OR “caregiver training”
Level 5	Bilingual	Bilingual* OR multilingual* OR “heritage language*” OR “first language” OR “home language” OR “community language” OR “culturally and linguistically diverse” OR CALD

For this reason, this study focused on identifying therapies that have included autistic children in environments where two or more first languages (L1s) are spoken in the household, or the home language (L1) differs from the societal language (L2). This allowed us to explore the specific language environments that have been studied, PLT implemented, and other patterns of demographic and outcomes reporting. While recent evidence indicates more research is needed to establish the effects of PLT in the monolingual setting (Conrad et al., 2021), the same must be held for the bilingual context. Indeed, it is arguably of greater imperative, given a) that bilingualism is now the global norm, b) the known obstacles to service accessibility for many bilingual families, and c) the difference in communication environments of bilingually exposed autistic children to those monolingually exposed.

Building on the findings of the pilot search, the following research question was developed to guide this review: What parent-led communication therapy programs available for families of autistic children have been examined in bilingual contexts? Additional research questions focused on identifying the language profiles of bilingual participants, how each study adapted the PLT to the bilingual context, and outcomes reported for both parents and children.

## Design

Scoping review methodology was used in five phases as per Khalil et al. (2016): 1) Identify research question, 2) identify relevant studies through a literature search, 3) select studies carefully, 4) extract and chart data in tabular and narrative format, and 5) collate results to identify implications. This scoping review allowed the research team to review the extent, range, and nature of PLT programs targeted to families of young autistic children as examined in bilingual language contexts, which can be used to guide discussions about supporting communication development of bilingual autistic children.

Given the existing evidence and clinical recommendations regarding the importance of early and specific support for autistic children, this study focuses on therapy programs targeting the early years, that is, children aged six years or less. Programs targeting broad communication skills were also included rather than focusing only on acquisition of core language skills such as vocabulary or grammar to include pre-communication skills, such as attention, initiation, and gesture. This total communication approach allowed for the consideration of different ways autistic children develop communication, including augmented and alternative communication (AAC) modes. Given the early years focus of this study, this approach also allowed for the inclusion of programs that included children who are pre- and/or minimally verbal.

## Methods

The protocol for this study was registered with the Open Science Framework (https://doi.org/10.17605/OSF.IO/HY9R2). This review was conducted in accordance with the JBI guidelines to ensure systematic and repeatable methodology (“JBI Manual for Evidence Synthesis,” 2020). This report is written in accordance with the Preferred Reporting Items for Systematic reviews and Meta-Analyses extension for Scoping Reviews (PRISMA-ScR) (Tricco et al., 2018) as provided for by the PRISMA 2020 statement of updated guidelines (Page et al., 2021). The PRISMA-ScR checklist is provided in Appendix A.

### Sources of Evidence Used as Eligibility Criteria

The span of a scoping review means that various evidence sources, both research and non-research, could be considered beyond what would be included in a systematic review (Peters et al., 2020).

Types of evidence included during searching were: 1) Primary research studies (quantitative and qualitative), including but not limited to case reports, case series, experimental studies, quasi-experimental studies, randomised control trials (RCT), and observational studies, 2) systematic reviews and 3) grey literature that was empirical in nature, including but not limited to conference papers, theses, and dissertations. Additionally, the reference lists of included papers were reviewed for appropriate references that met inclusion criteria.

Types of evidence excluded were: 1) Commentaries and opinion pieces, 2) evidence unavailable in either English or French, 3) full-text papers that could not be obtained using university access prior to the final analysis, and 4) papers without sufficient participant information to meet inclusion criteria.

As this review aims to map evidence in an emerging field of study, it was not anticipated that relevant papers would be found prior to 2010. However, a publication date limitation was set to 2003, as a 20-year window of research was considered reasonable to review.

### Search and Selection Methods

This search strategy was developed with the Faculty of Science and Medicine librarian at the University of Fribourg. The inclusion criteria and search terms were refined through pilot searching. Identified terms were searched at five levels for each database.

### Inclusion Criteria

This study used the following inclusion criteria:

1. Participants are parents/caregivers of children with a diagnosis of autism.
2. Autistic children were aged 6 years or younger.
3. Program targeted communication skills, including but not limited to language development, expressive and receptive communication, social communication, interaction behaviours, gestures, and pragmatic skills. Programs that targeted additional non-communication skills were included to the extent that communication outcomes were recorded.
4. Therapy was delivered by the parent(s). This criterion included therapy delivered in both coached and self-directed ways.
5. Participants lived in households in which two or more L1s are spoken OR the shared home language (L1) was different to the societal language (L2). For this criterion, it was not necessary for both parents to be bilingual, simply that they live in a bilingual household.

The concept of this study was to identify the availability of early support programs that can be delivered by parents and/or key caregivers to their autistic children regardless of their L1s. The context of this study was to identify PLT programs that have been examined in the bilingual environment. Programs could be offered face to face or virtually, and in an individual or group setting.

### Study Location and Selection

Searches were conducted using the following electronic databases: Medline–EBSCO, PsychINFO, ERIC, Web of Science. Empirical grey literature identified via Opengrey were considered if they met outlined inclusion criteria. Two searches were run in each database to ensure no papers were excluded at this stage. Search 1 included search terms at levels 1, 2, 3, and 4. This was repeated in Search 2 with level 5 terms added. All evidence was considered until 11 January 2023 (due to time and funding limitations) before screening commenced. Evidence published after this search date was not included in this review. All screening, selection, and full-text review took place within three months of this search date. The first author, SO, ran both searches and imported the titles and abstracts into Covidence (Kellermeyer et al., 2018). Reviewers shared access to collected papers for screening, selection, and extraction via Covidence. All reviewers were blinded to the decisions of others. All decisions were recorded on Covidence.

SO screened the title and abstracts of each located paper for inclusion or exclusion. KH, CH, MR, JZ, and SD each screened approximately 20% of papers, allowing for all collected evidence to be screened and selected by two reviewers independently. Full-text screening was conducted following the same process, with KH, CH, MR, and JZ each reviewing approximately 25% of selected papers, allowing for each paper to be reviewed by two reviewers independently. Conflicts and further questions were discussed and clarified until consensus was reached. There were no conflicts that were unresolved. Only papers with double agreement were selected for full-text review and included in this review.

### Data Extraction

An extraction template was developed in Covidence and used to extract data from the included studies. The data extraction tool was modified iteratively throughout this process. Alterations included additional prompts to facilitate the extraction of data related to key findings (parent and child outcomes) and limitations. SO extracted data from all papers. KH, CH, MR, and JZ extracted data from approximately 25% papers each. This process allowed each paper to be reviewed twice to ensure cross-checking and completeness of extraction. Conflicts and further questions were discussed and clarified until consensus was reached. There were no conflicts that were unresolved. Extractions were transferred to Excel spreadsheets. The following data were extracted from each included paper:

- Aims, publication year, methodology, sample size, country of study origin, language(s) included in study, caregivers included, age of child in question, parent and child outcome measures, and limitations.
- Details of the therapy program, including language(s) involved, service delivery type (in-person, virtual, individual, group), and types of measures used (quantitative, qualitative).

### Data Analysis and Presentation

Descriptive statistical analysis was conducted for data relating to study characteristics. Textual data on participant demographic information and reported outcomes were synthesised narratively using a descriptive qualitative content analysis approach (Peters et al., 2020). An inductive approach was used for synthesising and categorising data relating to participant information and outcomes, which was collaborative and iterative. This approach allowed for diverse positionality and reflexivity from investigators, adding to the trustworthiness of results obtained. SO created initial synthesis and categorisation of data, and all reviewers contributed to cross-checking and confirming summaries and results presented. The scoping review team discussed synthesis and presentation of data online. Review data are presented in tables, figures, and narrative summaries of findings relevant to the review aims and questions.

## Results

**Fig. 1:**
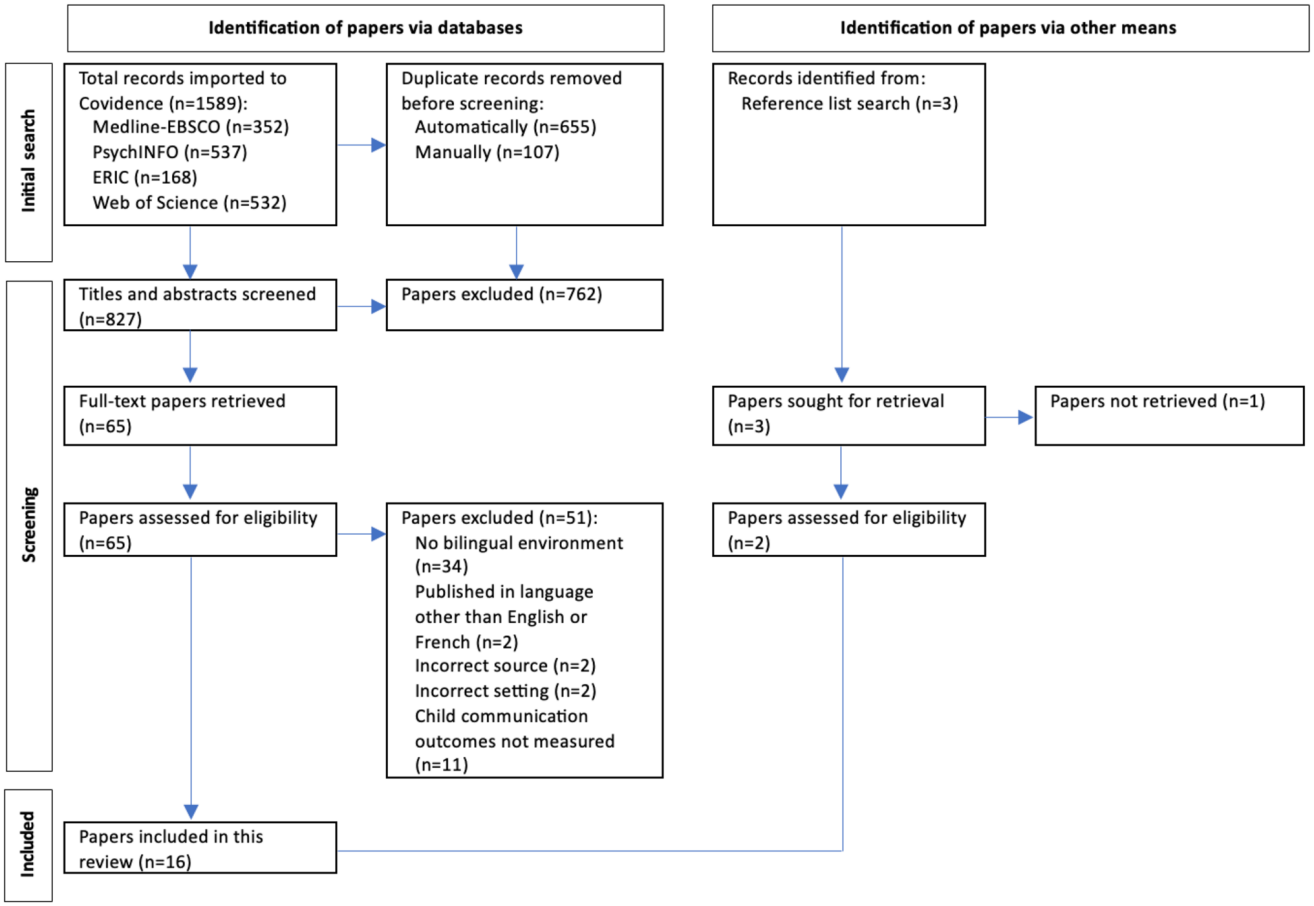
Search strategy

A search of five databases located 1645 papers, which were uploaded to Covidence. After duplicate citations were removed, a total of 827 papers remained for title and abstract screening. After excluding irrelevant articles (two of which may have been relevant but could not be accessed in either English or French), 65 full-text papers were retrieved and screened by two independent reviewers, which located 14 papers that met the eligibility criteria. After hand-screening the reference lists of eligible papers, an additional three papers were identified for screening. However, one could not be accessed in full text. In total, 16 papers met the eligibility criteria and were selected for final inclusion for this review. Figure 1 outlines the selection process and reasons for exclusion.

Of particular interest is the acceleration in studies reported since 2015, with all but one of the included papers published in the last eight years. Also of note is the lack of consistency in reporting on language environments for participants identified as bilingual. Of the 14 primary research studies (excluding the systematic review and research protocol), eight (57.1%) specified participants’ L1s, three (21.4%) reported that more than one language was spoken at home, one (7.1%) reported non-English L1, and two (14.3%) reported participants as simply bilingual. Of the eight that specified participant L1s, only two papers explicitly reported the language those participants used with their children, which was Spanish in both studies (Lopez et al., 2019; Meadan et al., 2020). Only two papers (14.3%) reported participant demographic data including age, place of birth, and socio-economic information (Lopez et al., 2019; Zeng et al., 2022).

### Characteristics of Included Studies

A summary of included papers is provided in Table 2. There was significant variation in the types of studies that were included in this review, as reflected in Table 3. Three-quarters of included papers were primary research articles (75.0%), with just under one third employing RCT design (31.3%), and over a third employing single-case experimental design (SCED) (37.5%) including multiple baseline design across participants. Of the studies involving participants (not including the research protocol and systematic review), almost half (42.3%) involved small sample sizes of fewer than 10 participants.

**Table 2:** Summary of papers included for review

Author/Year	Country of undertaking	Evidence source	PLT used	Aim	Design	Sample size	Service delivery	Participant L1 (*n*)	Participant L2 (*n*)	Lang. used in study
Abda et al., 2022	US	Dissertation	Pivotal Response Treatment (PRT)	Investigate the effectiveness of a 6-h training program in Pivotal Response Treatment (PRT) for parents of young children with ASD on the increased use of social functional utterances by their children during play sessions	SCED—multiple baselines	3 (fathers)	In-person	Arabic (3)	English	English
Bradshaw et al., 2017	US	Primary research article	Pivotal Response Treatment (PRT)	1. Conduct a preliminary investigation of the feasibility, effectiveness, and parent acceptability of a brief, parent-mediated intervention targeting verbal communication for toddlers exhibiting early symptoms of ASD. 2. Determine whether parent implementation of Pivotal Response Treatment (PRT) would result in changes in toddler verbal communication as measured by both the improvement in the frequency and quality of functional verbal communication as well as improvements on standardized tests	SCED—non-concurrent multiple baselines	3 (2 mothers, 1 parent)	In-person	Unspecified (1) English (2)	English	English
Brian et al., 2022a, 2022b	Canada	Primary research article	The Social ABCs (adapted)	Explore whether there were any differences between in-person and virtual delivery of the group-based program in terms of outcomes, feasibility, or acceptability	Multiple group comparison	82 (81 parents, 1 grand-mother)	In-person + virtual	Unspecified (21—most common Spanish, Italian, Tamil, Cantonese, Portuguese) English (61)	English	English
Brown & Woods, 2015	US	Primary research article	KidTalk-TaCTICS Project (KTTP)	Evaluate the effects of a parent-implemented communication intervention on parent and child communication for toddlers with Down syndrome, ASD, and developmental delays	SCED—non-concurrent multiple baselines	9 (mothers)	In-person	Luganda (1) English (8)	English	English
Coleman & Xu, 2020	US	Primary research article	Direct Trial Instruction (DTI) verbal mand intervention	Demonstrate a model for a parent-implemented DTI mand intervention intended to increase a child’s ability to verbally request for preferred items	SCED	1 (mother)	In-person	Unspecified (1)	English	English
Dodds, 2020	US	Research protocol	Helping Optimize Language Acquisition (HOLA)	1. Measure the effects of the HOLA intervention on parent knowledge of child development, parent PRT fidelity of implementation, and child social communication. 2. Measure parent satisfaction with the intervention and explore the impact of HOLA on parent stress, family empowerment, and child behavior. 3. Assess whether language has an impact on effectiveness of and satisfaction with HOLA	Single group pre/post study design	n/a	Virtual	Spanish (n/a)	English	Participants' choice
Elder et al., 2003	US	Primary research article	Overt social reciprocity training	1. Heighten father awareness of child interactions. 2. Evaluate the effects of an in-home intervention for autistic children with four culturally different father-child dyads	SCED—multiple baselines	4 (fathers)	In-person	Japanese (1) Spanish (1) English (2)	English	English
Ingersoll et al., 2016	US	Primary research article	ImPACT	Compare the effect of self-directed and therapist-assisted delivery models of ImPACT Online on key parent and child outcomes in preparation for a fully powered RCT	RCT	27 (parents)	In-person	Unspecified (NR)	English	English
Liao et al., 2023	Taiwan + US	Secondary research article (systematic review)	n/a	Review studies across cultures to summarize the characteristics of single-case studies on caregiver involvement in communication interventions for CLD families of individuals with ASD and IDD for recommendations on culturally responsive practices	n/a					
Lopez et al., 2019	US	Primary research article	Parents Taking Action (PTA)	Test the efficacy of a culturally tailored parent educational intervention for Latinx parents of children with ASD in a Southern California community	RCT	27 (mothers)	In-person	Spanish (22) English (5)	English	Participants' choice
Meadan et al., 2020	US	Program evaluation article	Parent-implemented Communication Strategies (PiCS)	Evaluate a translated and modified version of the PiCS program to support Spanish-speaking families with young children with autism and other developmental disabilities	SCED—multiple baselines	7 (mothers)	In-person + virtual	Spanish (7)	English	Participants' choice
Rahman et al., 2016	India + Pakistan	Primary research article	Parent- mediated intervention for Autism Spectrum disorder in South Asia (PASS)—adapted from Preschool Autism Communication Trial (PACT)	1. Assess the feasibility and acceptability of the parent-mediated intervention for autism spectrum disorder in South Asia in India and Pakistan	RCT	65 (parents)	In-person	Hindi (16), Urdu (35), English (6), Konkani (6), Marathi (2)	Marathi/Konkani (1), English/Konkani (1), Urdu (35), English (5), Konkani (6), Marathi (1),	Participants' choice
Rollins, 2018	US	Primary research article	Pathways Early Autism Intervention (Pathways)	1. Evaluate efficacy of Pathways in remediating the core deficits of sharing emotions in toddlers with ASD when compared to an intervention group without the innovative protocol and a BAU (business as usual) group. 2. Evaluate the efficacy of Pathways and the intervention without the IP in facilitating communication in toddlers with ASD when compared to a BAU group	RCT	34 (parents)	In-person	Spanish (one third) English (two thirds)	English	Participants' choice
Sengupta et al., 2021	India	Primary research article	Ummeed Parent Program for Autism (UPPA)—adapted from Improving Parents as Communication Teachers (ImPACT)	1. Examine if the content and structure of the intervention are acceptable to Indian parents. 2. Assess if Indian parents are able to implement the intervention. 3. Examine if parents observe any change in their children’s social-communication skills after the intervention. 4. Identify if participation in the intervention had any outcome on parental stress	Single group pre/post study design	114 (parents)	In-person	Unspecified (NR)	Hindi	Hindi
Shire et al., 2022	US	Primary research article	Joint Attention, Symbolic Play, Engagement and Regulation intervention (JASPER)	Explore strategies to support caregivers’ intervention implementation with their young children who are at various stages of their ASD diagnostic evaluations in a provincial public social service system where they are eligible for early intervention services	Multiple group comparison	56 (48 mothers, 6 fathers, 2 grandmothers	In-person	Unspecified (NR)	English	English
Zeng et al., 2022	US	Primary research article	Parents Taking Action (PTA)	1. Examine maintenance of treatment effects in a culturally tailored parent education program for Latinx families of children with autism spectrum disorder using a behavior maintenance framework	RCT	109 (mothers)	In-person	Spanish (93) English (16)	English	Participants' choice

**Table 3:** Description of characteristics of included papers

Study characteristics	*n*/16(%)	Sample characteristics	*n*/541 (%)
Type of evidence source		Caregiver who participated	
Experimental/quasi-experimental study	12 (75.0)	Parent	
Program evaluation	1 (6.3)	Mother	203 (37.5)
Research protocol	1 (6.3)	Father	13 (2.4)
Dissertation	1 (6.3)	Non-specified for gender	322 (59.5)
Systematic review	1 (6.3)	Grandmother	3 (0.6)
Region where the studies were conducted		L1 (home language)	
North America	13 (81.3)	Arabic (Libyan)	3 (0.6)
South Asia	2 (12.5)	English	118 (21.8)
Multiple regions	1 (6.3)	Hindi	130 (24.0)
Methodology used		Japanese	1 (0.2)
RCT^a^	5 (31.3)	Konkani	6 (1.1)
SCED^b^	6 (37.5)	Luganda	1 (0.2)
Single group pre/post study	2 (12.5)	Marathi	2 (0.4)
Multiple group comparison	2 (12.5)	Spanish	133 (24.6)
Systematic review	1 (6.3)	Urdu	35 (6.5)
Method of data collection		Not specified	112 (20.7)
Qualitative	0 (0)	L2 (societal language)	
Quantitative	6 (37.5)	English	363 (66.9)
Mixed/Multi-methods	10 (62.5)	Hindi	149 (27.5)
Average age of child in question		Urdu	30 (5.5)
< 1 year	0 (0)		
1–3 years	10 (62.5)		
4–6 years	5 (31.3)		
7 + years	0 (0)		
Not applicable	1 (6.3)		
Language of PLT delivery			
English	7 (43.7)		
Hindi	1 (6.3)		
Participants’ choice	6 (37.5)		
Not applicable	2 (12.5)		

^a^Randomised controlled trial

^b^Single case experimental design

Almost all the included studies were conducted in North American countries (87.5%), with the systematic review being undertaken between Taiwan- and the US-based institutions. No studies in this field have been published outside the North American or the South Asian subcontinent. Across all studies, mothers were the most represented caregivers reported (37.5%), with less than 1 percent of non-parent caregivers (grandmothers) reported (0.6%). However, more than half of participants were identified as gender neutral “parent” (59.5%). A broad array of L1s were reported.

**Fig. 2:**
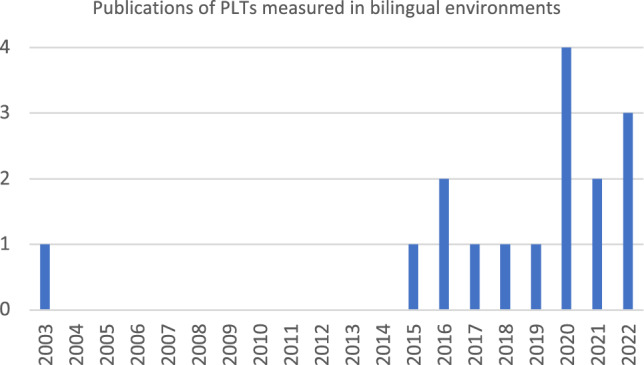
Timeline of publications of PLTs measured in bilingual environments

**Fig. 3:**
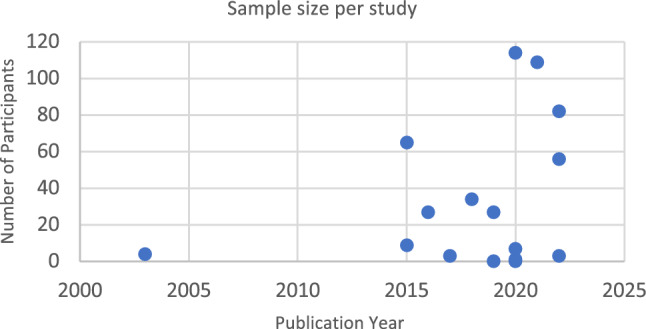
Timeline of sample size per publication

All but one of the included papers were published from 2015 (93.6%) (Fig. 2). Almost half of the studies involving participants sampled fewer than 10 participants (42.3%), with the only study published before 2015 including four fathers (Fig. 3).

Demographic information reported on participants was inconsistent. Ten studies reported participants’ caregiver role (mother, father, grandmother), and five simply reported that participants were parents of an autistic child. It is interesting to note that these five studies were published from 2019. Eight studies reported participants’ L1, with remaining studies reporting that participants were “bilingual”, spoke a “non-English language”, or spoke “languages other than English”. Spanish was the most reported L1, with two studies reporting on the country/culture of origin for those participants. This is unsurprising, given the large proportion of studies that were conducted in the US, where Spanish is the largest minority language spoken. The second largest L1 group reported was Hindi, however, participants were primarily from one study, which was conducted in India. Household income and marital status was reported by one study.

Three programs were adapted for virtual delivery (20%) (Brian, Solish, et al., 2022b; Dodds, 2020; Meadan et al., 2020). One study compared virtual and in-person delivery (6.7%) (Brian, Solish, et al., 2022b). One study compared self-directed and coached delivery (6.7%) (Ingersoll et al., 2016).

### PLT Programs for Autistic Children Undertaken in Bilingual Environments

A range of programs were utilised by the included papers (excluding the systematic review) to examine outcomes including bilingual participants, as are reflected in Table 4. All programs aimed to improve parents’ abilities to support their autistic children’s communication development, including focus on awareness of child development and child’s interactions (Dodds, 2020; Elder et al., 2003), social language during play (Abda, 2022), verbal language and social motivation (Bradshaw et al., 2017; Brown & Woods, 2015), functional requesting skills (Coleman & Xu, 2020), social communication (Brian, Solish, et al., 2022b; Ingersoll et al., 2016; Lopez et al., 2019; Meadan et al., 2020), and sharing emotions (Rollins, 2018).

**Table 4:** PLT programs and bilingual participants in included papers

Paper	PLT program	Total participants	Bilingual participants	Language asdaptations provided?	Outcomes reported mono- vs bilingually?
Abda, 2021	Pivotal Response Treatment (PRT)^a^	3	3	Yes	No
Bradshaw et al., 2017	PRT^a^	3	1	No	No
Brian et al., 2022a, 2022b	Social ABCs* (adapted)	82	21	No	No
Brown & Woods, 2015	KidTalk-TaCTICS Project (KTTP)(A merger of Enhanced Milieu Teaching (EMT)^a^ and Family-Guided Routines-Based Intervention (FGRBI)^a^)	9	1	No	No
Coleman & Xu, 2020	Direct Trial Intervention (DTI) verbal mand instruction	1	1	No	No
Dodds, 2020	Helping Optimize Language Acquisition (HOLA)	n/a – research protocol		Yes	n/a
Elder et al., 2003	Overt Social Reciprocity training (OSR)	4	1		No
Ingersoll et al., 2016	Improving Parents As Communication Teachers (ImPACT)^a^	27	Unspecified “other languages spoken in the home” (p.2277)	NR	No
Liao et al., 2023	n/a–systematic review				
Lopez et al., 2019	Parents Taking Action (PTA)^b^	27	22	Yes	No
Meadan et al., 2020	Parent-implemented Communication Strategies (PiCS)	7	7	Yes	No
Rahman et al., 2016	Parent- mediated intervention for autism spectrum disorder in South Asia (PASS) (adapted from Preschool Autism Communication Trial (PACT)^c^)	65	65	Yes	No
Rollins, 2018	Pathways early autism intervention (Pathways)^a^	34	Unspecified “a third” (p.21)	Yes	No
Sengupta et al., 2021	Ummeed parent program for autism (UPPA) (adapted from ImPACT^a^)	114	Unspecified (service delivered in Hindi, measures taken in English)	NR	No
Shire et al., 2022	Joint attention, symbolic play, engagement and regulation intervention (JASPER)^a^	56	10 (inferred from “17.86%” (p.657))	NR	No
Zeng et al., 2022	PTA^b^	109	93	Yes	No

^a^Program available as a clinical service provided by certified practitioners

^b^Broader than PLT to include psychoeducation, community advocacy, and other parent empowerment topics beyond support strategies training

^c^Clinically available as Paediatric Autism Communication Therapy (PACT)

An overall similarity identified between studies was the use of PLT programs that have been validated in monolingual English-speaking contexts. Only two programs that have been validated amongst English-speaking populations were adapted for CaLD populations of South Asia (13.3%) (Divan et al., 2015; Sengupta et al., 2021). Two programs were specifically designed for CaLD (Spanish L1) populations in the US (13.3%) (Dodds, 2020; Lopez et al., 2019). Five programs used in these papers that are publicly available as clinical services through certified practitioners are indicated in Table 4.

### Study Adaptations Made to Programs for Autistic Children in Bilingual Environments

Seven studies provided bilingual materials (6 English/Spanish, 1 English/Arabic), meaning that almost half of included studies (excluding the systematic review) provided, or intended to provide, written material in participants’ L1s (46.7%). Six studies provided in-person coaching in participants’ L1s where it differed from the societal language (40%), with two studies providing both written and in-person bilingual support (13.3%) (Dodds, 2020; Lopez et al., 2019). Three studies (20%) used or intended to use intermediaries (trained community workers from participants’ cultural groups who spoke the relevant heritage languages) to deliver the program in culturally and linguistically sensitive ways (Dodds, 2020; Lopez et al., 2019; Rahman et al., 2016).

Reporting of participant language profiles, language environments, and bilingual adaptations provided varied between studies. Some reported on specific demographic information for their sample groups including caregiver role, education level, household income, and heritage languages spoken by participants (Lopez et al., 2019; Meadan et al., 2020; Rahman et al., 2016; Zeng et al., 2022). These studies were focused on specific cultural and language communities and implemented culturally and linguistically tailored service delivery methods for their participants. Some studies broadly identified more than one language at home (Brian, Solish, et al., 2022b), bilingual parents (Coleman & Xu, 2020), families speaking multiple languages at home (Shire et al., 2022) and a parent who spoke a non-English language (Bradshaw et al., 2017). The PLT programs in these studies were delivered in English, and it is not clear how or if these studies adapted the materials to their bilingual participants. This is likely because the primary aim for many of these studies was not to examine impact within the bilingual context, and the inclusion of bilingual participants was incidental rather than a specific inclusion criterion.

### Outcomes for Parents and Autistic Children

Overall, included studies (excluding the research protocol and systematic review) examined feasibility, efficacy, and acceptability to parents in CaLD environments and reported positive outcomes for parents and their autistic children across a range of measures, as indicated in Table 5. Almost all studies reported increased parent fidelity of strategy implementation, with just one study reporting one out of four participants did not increase scores on this measure (Elder et al., 2003). This same study reported no change in one participant’s responsiveness to their child’s communication bids. Of the six studies that reported on changes in parent stress, four identified a decrease in stress, with Elder et al (2003) and Bradshaw et al (2017) reporting mixed results (one participant in each study reported no change in stress levels). However, all studies identified that the PLT programs conducted was acceptable and/or feasible to participants, who all reported overall satisfaction with the program in which they participated.

**Table 5:** Summary of parent and child outcomes

Paper	Parent outcomes					Child outcomes				
	Increased responsiveness to child communication?	Increased use of intervention strategies?	Decreased parent stress?	Acceptable/feasible?	Positive satisfaction?	Increased language/communication opportunities?	Increased expression of needs and wants?	Increased exchange of information?	Increased responses to parent communication?	Increased spontaneous language/communication?
Abda, 2021	Yes	Yes	NR	Yes	Yes	Yes	Yes	NR	Yes	Yes
Bradshaw et al., 2017	Yes	Yes	yes (2 out of 3)	Yes	Yes	Yes	Yes	yes	Yes	Yes
Brian et al., 2022a, 2022b	NR	NR	yes	Yes	Yes	NR	NR	NR	Yes	NR
Brown & Woods, 2015	Yes	Yes (8 out of 9)	NR	Yes	Yes	Yes	NR	NR	Yes	NR
Coleman & Xu, 2020	NR	Yes	NR	Yes	Yes	Yes	Yes	NR	Yes	Yes
Dodds, 2020	Research protocol									
Elder et al., 2003	Yes (3 out of 4)	Yes (3 out of 4)	Yes (3 out of 4)	Yes	Yes	NR	NR	NR	Yes	NR
Ingersoll et al., 2016	NR	Yes	Yes	Yes	Yes	Improvement in VAB-2^a^ scores				
Liao et al., 2023	Systematic review									
Lopez et al., 2019	Yes	Yes	Yes	Yes	Yes	Improvement in SCQ^b^ scores				
Meadan et al., 2020	Yes	Yes	NR	Yes	Yes	Yes	NR	Yes	Yes	NR
Rahman et al., 2016	Yes	Yes	NR	Yes	Yes	Improvement in VABS-2^a^ and MBCDI^c^ scores				
Rollins, 2018	Yes	Yes	NR	Yes	Yes	Yes	NR	NR	Yes	NR
Sengupta et al., 2021	Yes	NR	Yes	Yes	NR	NR	NR	NR	Yes	NR
Shire et al., 2022	NR	Yes	NR	Yes	NR	No	No	No	No	No
Zeng et al., 2022	NR	Yes	NR	Yes	Yes	NR	NR	NR	Yes	NR

*NR* Not reported

^a^Vineland Adaptive Behavior Scales-Second Edition

^b^Social Communication Questionnaire

^c^MacArthur-Bates Communicative Development Inventories

Almost all studies reported positive child outcomes, with only one study reporting no changes in communication outcomes measured (Shire et al., 2022). Positive communication outcomes reported include increased expression of needs and wants (Abda, 2022; Brian, Drmic, et al., 2022a; Coleman & Xu, 2020), and increased exchange of information (Bradshaw et al., 2017; Meadan et al., 2020). All studies except one reported the child’s increased response to parent-initiated communication. Of the five studies that reported on the child’s spontaneous communication, four reported an increase (Abda, 2022; Bradshaw et al., 2017; Coleman & Xu, 2020; Rahman et al., 2016). Three studies reported unspecified language and communication development through increases in standardised measures on the Vineland Adaptive Behavior Scales 2nd edition (VABS-2), Social Communication Questionnaire (SCQ), and MacArthur-Bates Communication Development Inventory (MBCDI) (Ingersoll et al., 2016; Lopez et al., 2019; Rahman et al., 2016).

Four studies used a control group (Lopez et al., 2019; Rahman et al., 2016; Rollins, 2018; Zeng et al., 2022), in which participants continued with “business as usual” therapy for their autistic children. These studies established pre-program equivalence of groups at baseline and reported on the effects of the PLT on primary outcome variables. Lopez et al (2019) reported improved SCQ scores from the PLT group post-program, while no significant change was reported from the control group over the same period. Increases in parent outcome measures were also significantly higher, including positive changes in depressive symptoms, which, while not statistically significant, were descriptively improved and nonetheless clinically meaningful. Rahman et al. (2016) reported that two out of three primary outcome measures (parent synchrony and child initiations) were significantly improved in the PLT group with large effect sizes, which were larger than those reported in the monolingual program from which this study had made CaLD adaptations. Rollins (2018) reported that the PLT groups had significantly better post-program performance on measures of social gaze, vocal/verbal reciprocity, and VABS-2 social scores on pragmatic diversity. Zeng et al. (2022) reported that participants in the PLT groups significantly improved in self-reported confidence in and frequency of using strategies. However, only one site out of two in this study reported improvements in family empowerment and child social communication measures. The differences between sites (different US states) were attributed to healthcare system and policy factors that differ widely in the US. It is worth noting that these four studies reported specific bilingual and culturally sensitive considerations for their bilingual participants when delivering the PLT.

Eight studies were single case experimental design, including case study, case series, and multiple baseline reports. In addition to overall positive outcomes on parent and child measures, excluding the research protocol, these studies reported additional benefits to PLT programs modified for linguistic and/or cultural sensitivity, including an increase in observed communication with other parent who did not speak the societal language (Abda, 2022), improved communication between siblings (Coleman & Xu, 2020), fathers taking on more childcare activities and a more direct childcare role in the household (Elder et al., 2003), and improved parent access to community and peer support systems (Meadan et al., 2020). Sengupta et al. (2021) found that responsive behaviours taught by their adapted PLT were readily incorporated in traditionally imperative, parent-dominated styles of family communication despite culturally-rooted discomfort with play-based strategies, once they realised the advantages of such an approach. Brown and Woods (2015) noted that variable parent strategy use resulted in variable child communication outcomes. These results indicate the particular benefit PLT can have in bilingual environments, as parents can use their L1s and generalise responsive communication support for their autistic children across daily settings.

Four studies compared groups of differing service delivery methods. Brian et al., (2022a, 2022b) reported no significant difference between virtual and in-person groups in primary outcomes of parent fidelity and child responsiveness. However, participants in the virtual group were observed to attend more sessions, and those in the in-person group were observed to complete more questionnaires. This study collapsed participants into groups of English- and non-English-speaking, and White and non-White ethnicity. Ingersoll et al (2016) reported that both self-directed and coached groups improved parent implementation fidelity and child standard scores, although the coached group made greater gains and were more likely to complete the program. However, there was no difference between these groups on measures of parent stress and self-efficacy. This study reported no adaptations made for their “minority status” participants. Meadan et al (2020), having delivered a program modified for the specific language group sample, reported participants preferred group rather than 1:1 service delivery. Shire et al (2022) reported no difference between groups provided with immediate coaching or coaching one month later. Not all participants disclosed their language environments at home and no linguistic adaptations were reported for those who did.

## Discussion

This scoping review aimed to map the available evidence on parent-led communication therapy that has been examined in the bilingual context for autistic children to identify the size and scope of current literature and formalise suspected gaps in the research body. Positive outcomes related to increased child responses to parent communication, increase in child spontaneous language, improvements in standardised measures, and parent satisfaction were reported across all studies. However, inconsistencies between demographic reports and a lack of specific data relating to language profiles of participants and their households make it difficult to empirically conclude on the efficacy of specific PLT programs studied for the bilingual context, and thus, all the more challenging to apply in the wider clinical context.

Despite the overall positive outcomes reported, it was impossible to identify specific results related to the bilingual participants and those from monolingual environments across all papers due to the variation in reporting between studies. This is likely because bilingualism and linguistic diversity were not part of the main aims for many included studies. Although it could be inferred that “business as usual” groups were English-language-only environments, none of the included papers used a specifically monolingual control group to compare outcomes with a bilingual group. Studies that specifically reported participants’ language profiles, language environments, and other demographic information related to cultural and linguistic heritage were those that targeted a limited geographic, cultural, or linguistic population. The studies that tailored PLT programs to their sample populations reported more nuanced outcomes, including advantages and disadvantages of service delivery mode and other obstacles to participation not currently addressed, as observed by their bilingual participants. It is recommended that more empirical study is conducted to examine how monolingually validated PLT programs function in bilingual contexts. For studies in which this is not a primary focus, it is recommended that studies including bilingual participants report more specifically on the nature of the bilingual environment involved. While detailed information on age of acquisition and summaries of interlocuters in each language setting may not always be feasible, specific information about levels of language exposure and which language(s) the participant is speaking with their autistic child would improve the interpretation of research results in the wider clinical and community context. It is also recommended that future research collects qualitative data during or after study implementation that investigate the extent to which PLT addressed the specific needs of families raising autistic children in bilingual environments, such as, discussion of conflicting advice received, issues of “dosage” of each language in the child’s environment, and matters of cultural identity and social communication in each language.

It is possible that recent shifts from reporting gender-specific caregiver roles are related to emerging evidence of a possible link between autism and gender dysphoria (Turban & van Schalkwyk, 2018), leading researchers to describe parents and caregivers in more gender-neutral ways. Understanding participants’ views of their gender roles within the cultural expectations of their families would be important, especially in cultures with strong values of traditionally understood “masculine” and “feminine” tasks and personas, which may directly affect the nature of communication environment of the child. Beyond mothers and fathers, many cultures have important values about family, where many family members, such as grandparents, aunts, and uncles, are key caregivers for children. Excluding these distinctions in research reporting risks overlooking the unique experiences, needs, and impact of significant carers in the child’s life. It is imperative that the richness of CaLD experiences is more clearly reported to better understand the communication environments in which CaLD autistic children are growing up. It would be of value for future studies to explore participant profiles further. In addition to investigating perceived gender roles within the family, valuable information would include questions such as, are they single, divorced, or step-parents? Are they neurodivergent themselves? What resources do they have available for such programs (time, childcare, devices and/or utilities for virtual modes of delivery, etc.)? These factors may inform the type of engagement parents demonstrate with PLT, which in turn may impact the effectiveness of such service delivery.

The use of virtual delivery in a telehealth format in some studies may have been in response to the recent SARS-CoV-2 pandemic. Beyond the pandemic, as research finds telehealth effective, practitioners will continue to utilise such technologies (Pacione, 2022; Rooks-Ellis et al., 2020). It is likely that future studies will continue to involve virtual sessions to further examine the outcomes for autistic children and their families in different modes of service delivery. It will be important for future researchers to consider the implications of telepractice for CaLD populations, While, on the one hand, it may increase a family’s access to practitioners who speak the parent’s L1, and remove barriers related to cost, time, and travel, it may, on the other hand, introduce barriers related to technology use, such as disparity of access to related resources (devices, stable internet, etc.).

Given that most of the available literature originates from the high-income North American context, with populations from largely middle-income socioeconomic groups, it will be important to explore acceptability and feasibility of monolingually, English-language validated PLT programs for historically marginalised groups from low-income CaLD backgrounds, as well as other populations in other parts of the world. It is imperative that CaLD adaptations are made, taking into account that culturally informed parenting styles may potentially conflict with the strategies taught in such PLT programs (Dubay et al., 2018; Joo & Liu, 2021). There has been some discussion from studies conducted in South Asia as to the acceptability and feasibility of modified PLT programs for a CaLD context for low- and middle-income countries. The feasibility of PLT among these populations is an important domain that warrants further empirical examination, including questions of whether parents have the resources to attend PLT programs without their children and whether virtual service delivery modes are acceptable for under-resourced families. This will demonstrate the impact of providing appropriate virtual PLT support for autistic children in CaLD environments, increasing service accessibility for vulnerable families.

Issues of accessibility and feasibility should be central to future studies exploring the implementation and impact of bilingual therapy delivered by parents in their L1(s). In outlining the evidence found, this scoping review has identified similarities and differences between sample populations that confirm suspected gaps in the literature relating to geographic and socioeconomic populations studied, and languages included. Given the absence of methodological quality appraisal, recommendations for practice will not be developed. However, conclusions may have relevance to practice and research reporting, and suggestions have been made based on these.

Our findings suggest that more specific questions for future systematic review involve in-depth appraisal of mixed/multi-method studies to develop recommendations for clinical practice and identify consistent reporting recommendations. Such review should be conducted to complement this scoping review, with the aim of examining the evidence from a smaller number of studies that may consequently serve to address a more specific research question. Suggestions for future primary research include feasibility and acceptability studies targeting populations from low socio-economic groups. This may include studies employing the use of, or providing materials in, participants’ L1s, exploring the impact on parent experience when speaking with an interpreter or versus speaking directly with practitioners, and examining whether the training of community support workers who may act as CaLD intermediaries between parents and practitioners can be extended to training interpreters in order to enhance the experience of families of autistic children participating in PLT. On a greater systems level, it would be valuable for future research to investigate solutions to address issues of access to bilingual clinicians, CaLD community support workers, and interpreters for families as well as healthcare and educational facilities. It is also recommended that future studies measure outcomes in terms of bilingual and monolingual environments, particularly in the context of autism and PLT designed to improve communication outcomes for young children. Relevant research questions may include: To what extent do, or could, current PLT programs incorporate a focus on improving bilingual opportunities for autistic children? What strategies are, or could, be useful in supporting bilingualism and bilingual proficiency for autistic children? What do CaLD families identify as important goals for their autistic children, and do they/how do they differ from monolingual families? More qualitative exploration is needed regarding the values, perspectives, and preferences of bilingual families from non-“Western”, CaLD, and low-resource backgrounds to interrogate the extent to which the strategies offered by current PLT programs fit families’ cultural perspectives and expectations, and what support such parents may prefer. It will also be important that future research considers the strengths and skills of such families, especially family structures with multiple key carers beyond parents, and how these can be incorporated into PLT programs when creating specialised support plans for CaLD autistic children.

## Limitations

Given that this is a scoping review and not a systematic review, the methodology of each included study and effectiveness of PLT programs delivered to bilingual parents of autistic children have not been qualitatively evaluated. However, broad results have been obtained and the size and scope of available evidence has been identified. In the absence of methodological quality appraisal, an in-depth analysis of the literature and specific PLT programs was not conducted and recommendations for practice could thus not be developed. Nevertheless, these conclusions are arguably relevant to clinical practice and recommendations for future research have been made.

A limitation to this scoping review is the reduced search for gray literature, which did not include the database ProQuest. Given the finding that a large majority of research on this topic has come from North American institutions, it is likely that a North American-based database, such as ProQuest, rather than European-based Opengrey, would have yielded more papers (theses and/or dissertations) for inclusion in this review. However, more outcomes from North American institutions including North American populations would only strengthen the conclusion that evidence in this specific field is largely North American in context.

Due to time and funding constraints, source databases were searched on one date before screening commenced, and all screening was completed within 3 months of this date. Evidence published following this search date is not considered by this scoping review and should be taken into account in future reviews.

A limitation of this protocol is the exclusion of evidence that is not available in English or French, which are both western, European-based languages. This may increase the possibility of cultural bias. We excluded articles we could not read in German and Spanish. Should these papers be relevant to our eligibility criteria, this would impact our results regarding origin of studies completed in this field.

Our scoping review specifically explored PLTs to the exclusion of other forms of early care programs targeted at autistic children. As an emerging field of study, it is important to examine such approaches to support autistic children in their early years of development, particularly when PLT may address issues of access to resources by bilingual families that have already been raised in the literature. Although scoping reviews allow for wide data collection, the researchers did not want to dilute key findings of this study by having an extremely expansive subject area. Further reviews may explore the evidence targeting other areas not included in the scope of this review, such as school-based therapies, programs targeting developmentally older children, or programs that centre on different developmental “disorders” besides autism.

## Conclusions

This scoping review has highlighted the paucity of bilingual PLT programs studied for children on the autism spectrum that target bilingual participants in specifically bilingual environments. Language profiles of participants and the language environments of autistic children must be specifically reported. Despite the surge of research conducted in the past few years, more examination, both quantitative and qualitative, is needed to validate the emerging results and better understand the impacts and outcomes of such support for families of children on the spectrum in complex situations beyond traditional monolingual norms.

## Footnotes

1 Kenny et al. (2016) investigated a range of UK stakeholders’ perspectives on the terminology and found that ‘autistic’ was endorsed by a large percentage of autistic adults, family members/friends, and parents. In a similar study conducted in Australia, Bury et al. (2023) reported ‘autistic’, ‘person on the autism spectrum’, and ‘autistic person’ as most preferred and least offensive overall. Given these findings, this study uses these terms interchangeably.

2 Bilingualism is defined in this study as the use of two or more languages in everyday life (Grosjean, 2010). This definition, with a focus on language use rather than specific fluency markers, is deemed most appropriate in this context as autistic children experience difficulties with language and communication behaviours, whether mono- or bilingually exposed.

3 Considering recent research, this study will privilege terminology to indicate ‘specific support’ or “service’ rather than “intervention” or “treatment” to respect autistic-preferred language (Monk et al., 2022).
